# A case presentation of an IgA nephropathy patient with Vogt-Koyanagi-Harada syndrome

**DOI:** 10.1186/s12882-020-01938-y

**Published:** 2020-07-13

**Authors:** Quan Zhang, Xing Fan, Meng Tian, Hongling Han

**Affiliations:** 1 Department of Nephrology, Tianjin Medical University General Hospital, Tianjin, China; 2 School of Graduate, Tianjin Medical University, Tianjin, China; 3 Department of Nephrology, Tianjin 4th Central Hospital, Tianjin, China; 4 Tianjin Jinnan Hospital, Tianjin, China

**Keywords:** IgA nephropathy, Vogt-Koyanagi-Harada syndrome, Human leukocyte antigen, Case presentation

## Abstract

**Background:**

Vogt-Koyanagi-Harada syndrome is a rare disease characterized by skin and eyelash bleaching, chronic granulomatous iridocyclitis and exudative retinal detachment, and aseptic meningitis and encephalopathy. IgA nephropathy complicated by Vogt-Koyanagi-Harada syndrome is very rare, even though they might have similar pathogeneses. Ocular lesions often are not examined when patients are diagnosed with IgA nephropathy, which affects the prognosis.

**Case presentation:**

We describe a 55-year-old male IgA nephropathy patient who was admitted with high fever and hematuria. Physical examination revealed impaired binocular vision with blurred vision, impaired hearing, and a congestive rash on the chest and back. Renal ultrasound examination showed no abnormalities. Laboratory examination showed that glomerulonephritis was complicated by infection, and anti-infection therapy was ineffective. Bilateral fluorescein angiography showed Vogt-Koyanagi-Harada syndrome. Further renal biopsy confirmed IgA nephropathy. Hormone shock therapy and cyclophosphamide adjuvant therapy were administered, and the patient’s symptoms improved.

**Conclusion:**

For the first time, we reported the case of simultaneous onset of IgA nephropathy and Vogt-Koyanagi-Harada syndrome, which is very rare. The onset of Vogt-Koyanagi-Harada syndrome is rapid and serious, while that of IgA nephropathy is relatively milder, making it easy for specialized doctors to neglect this condition. Doctors should be highly alert to the clinical concomitant occurrence of the two diseases with similar mechanisms, especially in the case of neurological defects and ocular symptoms in IgA nephropathy patients, since timely immunosuppressive treatment may improve the outcome of ocular diseases.

## Background

IgA nephropathy (IgAN) is common worldwide, especially in China. It is characterized by the varying extent of mesangial proliferation and mesangial immune complex deposition, with IgA as the predominant or codominant immunoglobulin type [1]. IgAN is restricted to the kidneys in most cases; however, it is sometimes accompanied by other conditions, such as ankylosing spondylitis, celiac disease, alcoholic and nonalcoholic liver disease, sarcoidosis, and dermatitis herpetiformis. The most common concomitant injury of the eyes in IgAN patients is scleritis, but ocular involvement is infrequent.

Vogt-Koyanagi-Harada syndrome (VKHS) is an inflammatory disease resulting in damage to multiple systems in the body, including granulomatous uveitis and meningeal stimulation, with or without auditory dysfunction, skin or hair changes [2]. VKHS and IgAN might have similar pathogeneses, but cases of VKHS combined with IgAN are rarely reported. We encountered one patient who developed VKHS with IgAN at the same time.

## Case presentation

A 55-year-old man was admitted to our department with fever for 10 days and gross hematuria for 9 days. The patient had developed fever with a maximum temperature of 39 °C, without chills, abdominal pain or diarrhea 10 days prior. The temperature returned to normal after antipyretic and antibiotic administration under the guidance of community doctors, but it increased again and was accompanied by gross hematuria just a few hours later. The patient gradually developed foamy urine with eye pain, photophobia, tinnitus, rash and other discomforts before this recent admission to our department.

Physical examination revealed increased temperature (37.7 °C), normal blood pressure (Bp, 100/80 mmHg), and bradycardia (HR, 56 bpm). There was hyperemia on his head, neck and chest skin and hyperemia and rash on his back. His physical exam was otherwise unremarkable. Specifically, he had no oral lesions, lymphadenopathy, joint swelling, lower extremity edema, or cardiac murmur or rub.

A history of renal lithiasis for 3 years could be elicited. The diagnosis of renal lithiasis was established at the local hospital, and the patient had received extracorporeal lithotripsy at the same hospital. The patient denied eye trauma and a history of surgery. He was unaware of any familial genetic disease and had not been tested for a genetic disease.

The results of the initial laboratory tests were as follows: white blood cell count 15.0 × 109/L, neutrophil ratio 86.3%, hemoglobin 118 g/L, platelets 215 × 109/L; albumin 24 g/L, blood urea nitrogen 6.4 μmol/L, plasma creatinine 79 μmol/L, uric acid 130 μmol/L. blood sugar 5.8 μmol/L. potassium 4.67 μmol/L, sodium 142 μmol/L, chloride 104 μmol/L, calcium 2.11 μmol/L, and CO_2_CP 25 μmol/L. Urine erythrocytes (6250.00 μL) and urinalysis showed 3+ blood and 1+ protein with 8–10 white blood cells/high-power field. Twenty-four-hour urine collection revealed 0.673 g of protein. Tests for the presence of rheumatoid factor, ANA and ANCA were negative. Tests for the presence of anti-mitochondrial and anti-smooth-muscle antibodies were negative. Complement levels were normal.

**Fig. 1:**
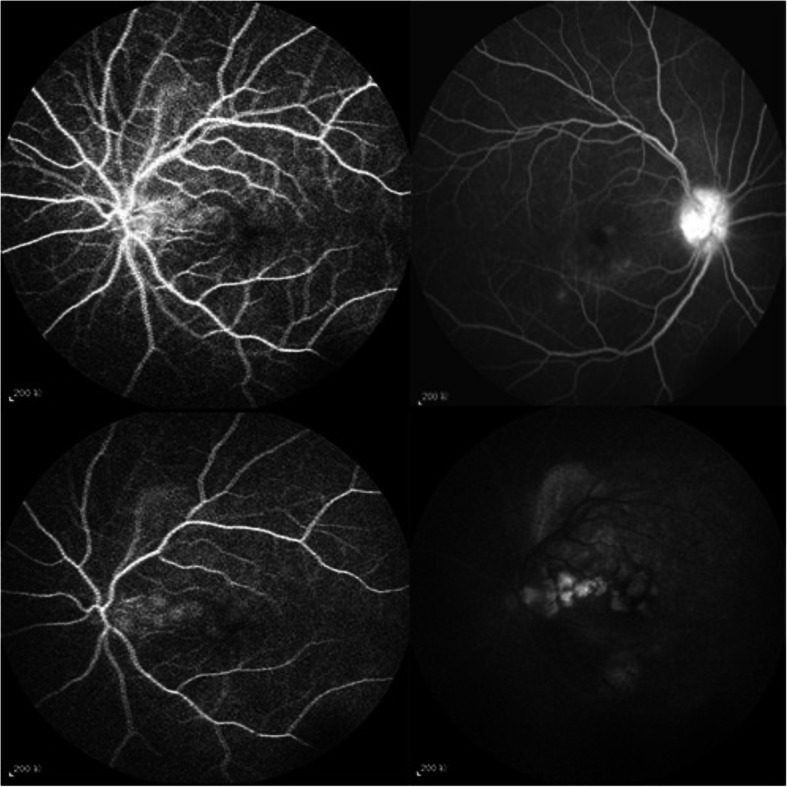
Fluorescein angiography revealed multiple leakages from the optic discs as well as the retinal pigment epithelium in the posterior pole of both eyes and the accumulation of cystic fluorescein

Symptoms gradually worsened after admission, and the body temperature fluctuated at 39–41 °C with more hematuria and more extensive rash. In addition, the patient developed a severe headache, neck stiffness, eye pain, conjunctival congestion, and decreased vision in both eyes, accompanied by tinnitus and hearing loss. The hearing test showed severe neurological deafness in both ears. The slit lamp examination showed that the conjunctiva of both eyes was mixed and congested, and mutton-fat-like KPs were observed, but the cornea was still clear. Bilateral intraocular pressure increased to 30 mmHg in the left and 35 mmHg in the right. Fluorescein angiography revealed multiple leakages from the optic discs as well as the retinal pigment epithelium in the posterior pole of both eyes and the accumulation of cystic fluorescein, typical of Vogt-Koyanagi-Harada syndrome, as shown in Fig. 1.

**Fig. 2:**
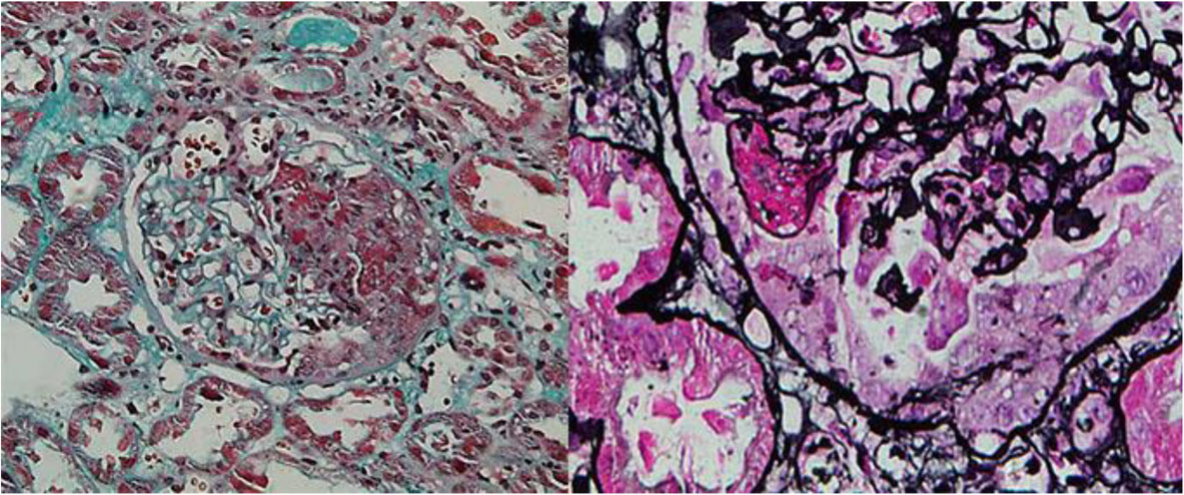
Renal biopsy showed 17 glomeruli, among which 2 small cellular crescent bodies with fibrinoid necrosis were observed as well as renal interstitial fibrosis. Immunofluorescence indicated IgA+++, IgG+, IgM-, C3++, C1q-, and FRA++ deposits along the glomeruli, consistent with IgA nephropathy with focal hyperplasia and necrosis

A percutaneous renal biopsy was then performed. Sampling consisted of 17 glomeruli with 2 small cellular crescents with fibrinoid necrosis and 2 ischemic sclerosis and fuchsinophilic protein deposition in the mesangial area. Lymphocytes and monocytes showed multifocal and flaky infiltration in the renal interstitium with fibrosis. Immunofluorescent examination revealed strong IgA (3+) deposition along the glomeruli, as well as IgG(+), IgM(−), C3(++), C1q(−), and FRA(++). Based on the findings mentioned above, the diagnosis of IgA nephropathy (M1E0S1T1C1) with VKHS was confirmed, as shown in Fig. 2.

During hospitalization, the patient was given a bolus of methylprednisolone 500 mg 3 times, followed by treatment with prednisone 60 mg, mannitol and cyclophosphamide. The patient’s symptoms were significantly relieved, intraocular pressure was reduced to normal, urinary protein was reduced to 208 mg/24 h, and red blood cells were reduced to 1.90 μL by contrast microscopy.

The patient is still taking prednisone and intermittent cyclophosphamide treatment, the urine test results have returned to normal and his condition is stable, without complaints of blurred vision or headache.

## Discussion and conclusion

Vogt-Koyanagi-Harada syndrome is a rare multisystemic disease that affects tissues containing melanin, such as the eye, inner ear, meninges, and skin [3]. VKHS is predominantly found in patients aged between 20 and 50 years. The disease occurs more frequently among Asian, Native American, and Hispanic populations. VKHS is one of the most common causes of uveitis in Japan and Brazil [2]. Although the exact etiology of VKHS remains unclear, many studies have suggested that it is associated with genetics and the autoimmune system. VKHS is thought to be a T-cell-mediated autoimmune process directed against one or more antigenic components of melanocytes. CD4+ T cells, Th1 cytokines and HLA-DRB1*0405 play the main role in the pathogenesis of VKHS [4, 5]. Previous reports have shown that CD4+ T cells sensitive to melanocytes are responsible for the development of VKHS [6]. Viruses, such as the Epstein–Barr virus and cytomegalovirus, have been hypothesized as possible triggering factors of these mechanisms [7]. The disease is characterized by chronic bilateral panuveitis associated with a varying constellation of auditory, neurological and cutaneous manifestations [8]. The classic clinical course of VKHS can be divided into 4 stages: the prodromal stage, the acute uveitis stage, the convalescence stage, and the chronic recurrent stage in some patients [8].

Previous studies have found that VKHS can also be associated with other autoimmune disorders, such as ulcerative colitis, Crohn’s disease, IgAN, autoimmune polyglandular syndrome type 1, hypothyroidism, diabetes mellitus, and Hashimoto’s thyroiditis. As far as we know, only 2 cases of IgA nephropathy complicated by this syndrome were reported in Japan in 2007 [9]. At the initial diagnosis of IgA nephropathy by renal biopsy, one patient showed low disease activity with low-grade mesangial cell proliferation, without hormone therapy, and VKHS appeared 11 years later. The other patient presented high pathological activity with glomerular crescent formation, which was treated with pulse corticosteroid therapy with oral prednisolone tapering, and presented the syndrome again 5 years later. IgA nephropathy was stable in both patients without corticosteroid treatment at the time of eye disease presentation.

However, in our case, the patient had no previous extrarenal manifestations of IgAN or discomfort with eye diseases and presented hematuria, proteinuria and fever at the onset, accompanied by discomforts such as blurred vision and hearing loss. The renal manifestations and systemic lesions occurred at the same time, and the pathological manifestations were highly active. In consideration of the patient’s condition, pulse corticosteroid therapy and steroid tapering were administered, with the addition of cyclophosphamide treatment. After treatment, the patient’s vision and hearing were restored, and the symptoms of renal damage were reduced, which was consistent with previous literature reports [10].

Interestingly, our patient represents the first case diagnosed with both VKHS and severe IgAN, which may or may not be an incidental association. We believe that immunological mechanisms probably play an important role in this association.

Both Vogt-Koyanagi-Harada syndrome and IgA nephropathy are well known to be sometimes preceded by common cold-like symptoms. Virus infection (EB, CMV) may be involved in both diseases simultaneously as a trigger of further abnormal immune mechanisms [11]. VKHS is a systemic disease with an abnormal immune response of T cells. CD4+ T cells, T helper 17 (Th17) cells and regulatory T (Treg) cells play an important role in VKHS activities. The activation of C3aR in CD4+ T cells can upregulate the production of inflammatory factors such as IL-17, and the increased expression of C3aR may also lead to the overactivation of the Th17 cell response and promote the occurrence and development of VKHS [12]. However, the dysregulation of CD4+ T cell subsets also participates in the pathogenesis of IgAN. IgAN is characterized by higher proportions of circulatory Th2, Th17, and Th22 cells but lower proportions of Th1 and Treg cells. An imbalance of the ratio of Treg cells to Th17 cells is related to the pathogenesis of IgAN and is correlated with clinical severity. In IgAN, IL-4 (Th2 type IL), IL-17 (Th17 type IL) and TGF-β may inhibit the expression of C1GALT1 and its chaperone (C1GALT1C1), which is mainly responsible for the addition of N-acetylgalactosamine (GalNAc) to serine or threonine in the hinge region. The dysfunction of this process is an important mechanism for the formation of glycosylated abnormal IgA1 [13].

More incredibly, both diseases show immunological abnormalities and have genetic predispositions, such as familial occurrence and strong association with human leukocyte antigens (HLAs). IgAN is closely related to the HLA-DQB1, HLA-DRB1, HLA-DP, and DEFA alleles and shares most alleles with other immune system diseases, including Crohn’s disease, ulcerative colitis, and SLE [14]. Coincidentally, VKHS is also closely related to these diseases, and HLA-DRB1*0405 is also the main susceptibility allele of VKHS [15]. These findings suggest that there may be an internal link between the two diseases that is still unclear and needs to be explored further.

For the first time, we reported the case of simultaneous onset of IgAN and VKHS, and our data supplement those of previous cases. The onset of VKHS is rapid and serious, while that of IgAN is relatively milder, making it easy for specialized doctors to neglect this condition. Doctors should be highly alert to the clinical concomitant occurrence of two diseases with similar mechanisms, especially in the case of neurological defects and ocular symptoms in IgAN patients, since timely immunosuppressive treatment may improve the outcome of ocular diseases.

## Data Availability

All data generated or analyzed during this study are included in this published article.

## Abbreviations

VKHS: Vogt-Koyanagi-Harada syndrome
IgAN: IgA nephropathy
HLA: Human leukocyte antigens
